# Using computed tomography angiography and computational fluid dynamics to study aortic coarctation in different arch morphologies

**DOI:** 10.3389/fped.2023.1131025

**Published:** 2023-06-27

**Authors:** Jinjie Qin, Da Ouyang, Taocui Yan, Haoru Wang, Kui Guo, Xin Jin, Zhengxia Pan, Ling He

**Affiliations:** ^1^ Department of Radiology, Children’s Hospital of Chongqing Medical University, National Clinical Research Center for Child Health and Disorders, Ministry of Education Key Laboratory of Child Development and Disorders, Chongqing Key Laboratory of Pediatrics, Chongqing, China; ^2^ Department of Cardiology, Children’s Hospital of Chongqing Medical University, National Clinical Research Center for Child Health and Disorders, Ministry of Education Key Laboratory of Child Development and Disorders, Chongqing Key Laboratory of Pediatrics, Chongqing, China; ^3^ Medical Data Science Academy, College of Medical Informatics, Chongqing Medical University, Chongqing, China; ^4^ Department of Cardiothoracic Surgery, Children's Hospital of Chongqing Medical University, National Clinical Research Center for Child Health and Disorders, Ministry of Education Key Laboratory of Child Development and Disorders, Chongqing Key Laboratory of Pediatrics, Chongqing, China

**Keywords:** aortic coarctation (CoA), gothic arch, computational fluid dynamics (CFD), AAO-DAO angle, computed tomography angiography (CTA)

## Abstract

**Objective:**

To study the differences in computed tomography angiography (CTA) imaging of gothic arches, crenel arches, and romanesque arches in children with Aortic Coarctation (CoA), and to apply computational fluid dynamics (CFD) to study hemodynamic changes in CoA children with gothic arch aorta.

**Methods:**

The case data and CTA data of children diagnosed with CoA (95 cases) in our hospital were retrospectively collected, and the morphology of the aortic arch in the children was defined as gothic arch (*n* = 27), crenel arch (*n* = 25) and romanesque arch (*n* = 43). The three groups were compared with D1/AOA, D2/AOA, D3/AOA, D4/AOA, D5/AOA, and AAO-DAO angle, TAO-DAO angle, and aortic arch height to width ratio (A/T). Computational fluid dynamics was applied to assess hemodynamic changes in children with gothic arches.

**Results:**

There were no significant differences between D1/AOA and D2/AOA among gothic arch, crenel arch, and romanesque arch (*P* > 0.05). The differences in D3/AOA, D4/AOA, and D5/AOA among the three groups were statistically significant (*P* < 0.05), D4/AOA, D5/AOA of the gothic arch group were smaller than the crenel arch group, and the D3/AOA and D5/AOA of the gothic arch group were smaller than the romanesque arch group (*P* < 0.05). The difference in AAO-DAO angle among the three groups was statistically significant (*P* < 0.05), and the AAO-DAO angle of gothic arch was smaller than that of romanesque arch and crenel arch group (*P* < 0.05). There was no significant difference in the TAO-DAO angle between the three groups (*P* > 0.05). The difference in A/T values among the three groups was statistically significant (*P* < 0.05), and the A/T values: gothic arch > romanesque arch > crenel arch (*P* < 0.05). The CFD calculation of children with gothic arch showed that the pressure drop between the distal stenosis and the descending aorta was 58 mmHg, and the flow rate at the isthmus and descending aorta was high and turbulent.

**Conclusion:**

Gothic aortic arch is common in CoA, it may put adverse effects on the development of the aortic isthmus and descending aorta, and its A/T value and AAO-DAO angle are high. CFD could assess hemodynamic changes in CoA.

## 1. Introduction

Aortic coarctation (CoA) refers to a certain degree of narrowing of the aortic arch caused by various causes. It causes high blood pressure in the proximal end of stenosis, low blood pressure at the distal end, congestive heart failure, refractory hypertension, etc. It is one of the most common complex congenital heart diseases in children (1–3). At present, the disease mostly relies on transthoracic echocardiography (TTE), computed tomography angiography (CTA), and cardiac magnetic resonance (CMR) for morphological diagnosis, and reflects the development of the aortic arch by measuring the diameter of each level of the aortic arch and then calculating the proportion (4, 5).

The treatment of CoA requires restoring aortic anatomy as much as possible, thereby lowering systolic blood pressure in the upper extremities and increasing blood perfusion in the lower extremities. However, children with CoA could develop a specific aortic arch morphology. For example, in Turner Syndrome (TS), the aortic arch morphology often presents with an elongated transverse arch and a flattened and twisted arch (6). Studies have shown that this arch morphology is at great risk of hypertension and ascending aortic dilation in patients with TS, and it is recommended to include an evaluation of aortic arch morphology in the hypertensive risk stratification of TS (7). The shape of the aortic arch can be described as Gothic, Crenel, and Romanesque (8), derived from the corresponding architectural style. The Gothic arch is triangular, with an acute angle between the ascending and descending aorta, and the transverse arch of the aorta is shortened or absent; the Crenel arch is rectangular, and the length of the aortic transverse arch is normal; the Romanesque arch is somewhere in between, round (9). Whether different arch morphologies have an effect on the morphological development and hemodynamic changes of the aortic arch has not been confirmed by relevant studies. At the same time, Computational fluid dynamics (CFD), as a medical-engineering combination technology, could calculate intravascular hemodynamic indicators based on images (10, 11), and has made certain research progress in the diagnosis, treatment, and follow-up of intracranial aneurysm, aortic dissection, atherosclerosis, and other vascular diseases. Therefore, this study aims to evaluate the morphology and arch development of aortic arches in CoA patients and apply CFD to describe the kinetic significance of special aortic arch morphology in patients with CoA, so as to provide more powerful clinical references.

## 2. Materials and methods

### 2.1. General data

Retrospectively collected 95 children with CoA in our hospital from March 2017 to October 2022, 54 males and 41 females, aged 0.03–77.43 months. The median age was 3.00 months. Patients could be included when they met the following conditions: (1) patients received both chest CTA and TTE confirmed CoA within 2 weeks prior to surgical correction; (2) patients excluded aortic arch branch development variant, transposition of the great arteries (TGA), interrupted aortic arch (IAA), and aortitis and other aorta-related diseases. This study is retrospective and has been reviewed by the Ethics Committee of Children's Hospital Affiliated to Chongqing Medical University (No. 394 of 2021).

### 2.2. Echocardiography

GE Vivid E9 (General Electric, USA) and Philips iE 33 (Philips, Netherlands) multifunctional ultrasound diagnostic instruments, probe frequencies of 1.7–3.3 and 1–5 MHz. Record Peak Systolic Pressure Gradient (PSPG) at the aortic arch across stenosis.

### 2.3. CTA scanning

The patients underwent CTA scan with Philips Brilliance iCT at the initial diagnosis. Dexmedetomidine nasal sedation was performed by the anesthesiology department for children who could not cooperate. The scan ranged from the lower neck to the diaphragm level. The tube voltage was 80–100 kV, the tube current was automatically adjusted, the acquisition slice thickness was 5.0 mm, the pitch was 1.1 mm, the collimation was 0.6 mm, and the reconstruction slice thickness was 1.0 mm. The contrast scan was performed respectively at 15–30 and 50–60 s by hand or dorsal foot venously injected iodine contrast medium (320 mg/L, 1.5–2.0 ml/kg, and a flow rate of 0.5–3.5 ml/s).

### 2.4. CTA image analysis

**Figure 1:**
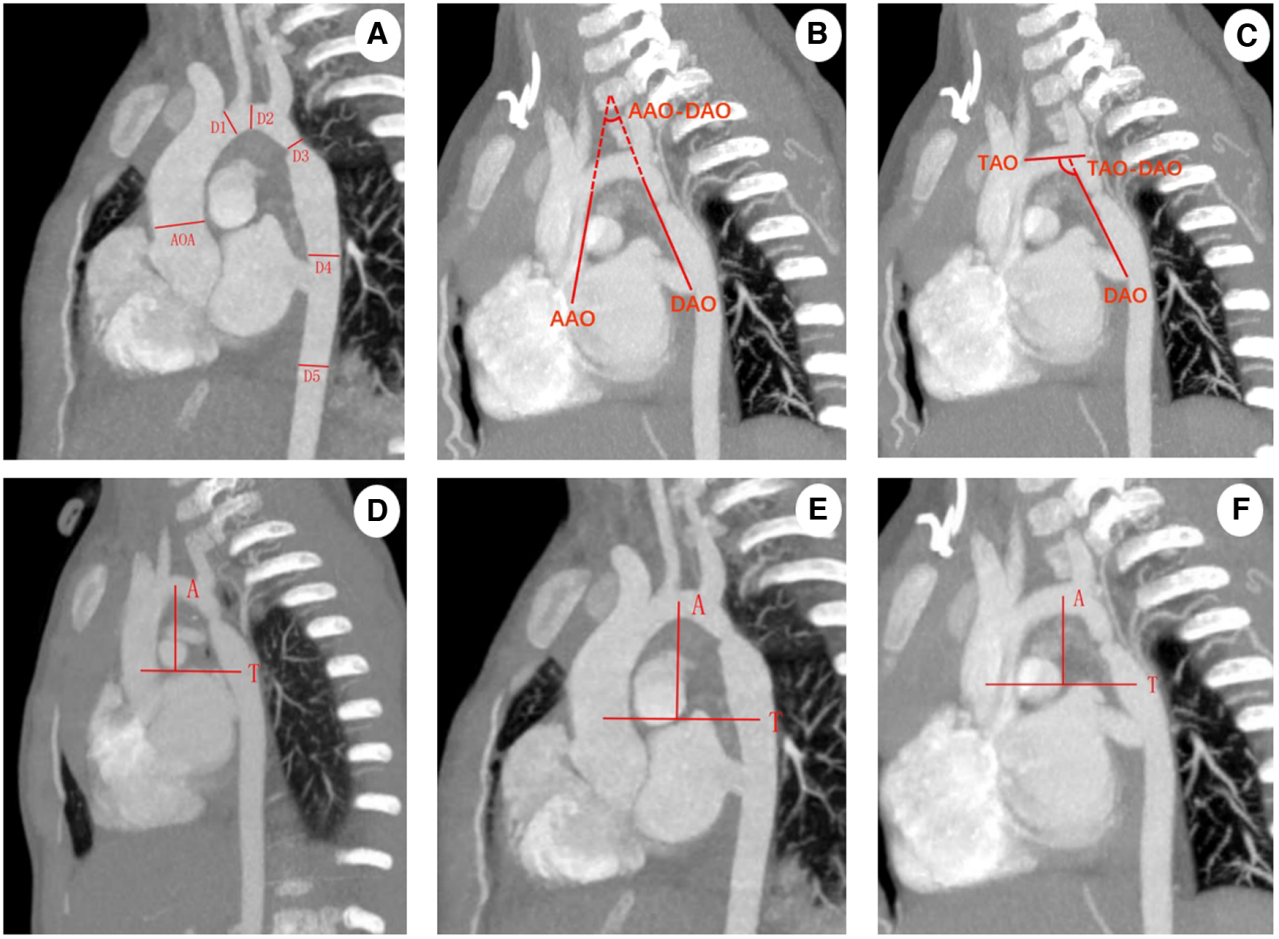
(**A**) Oblique sagittal MIP image measurement, AOA: maximum inner diameter of the ascending aorta; D1: maximum inner diameter of the proximal arch; D2: maximum inner diameter of the distal arch; D3: maximum inner diameter of the isthmus; D4: the middle section of the descending aorta; D5: maximum inner diameter of the descending aorta at the transverse septum; (**B,C**) oblique sagittal position MIP image measurement, AAO-DAO angle and the TAO-DAO angle are the angle between straight line AAO and DAO, TAO and DAO, respectively; AAO: a straight line connecting AAO to the anterior border of the left brachiocephalic trunk artery along the internal border of the ascending aorta; DAO: a straight line connecting the descending aorta to the posterior border of the left subclavian artery (LSCA) along the internal border of the descending aorta; TAO: a straight line connecting the anterior border of the left brachiocephalic trunk artery to the posterior border of the LSCA along the internal border of the transverse arch of the aorta; (**D–F**) aortic arch height to width ratio measurement: aortic arch width (T) refers to the maximum horizontal distance between the end point of the ascending aorta and the midpoint of the descending aorta, aortic arch height (A) refers to the vertical distance from the highest point of the aortic arch centerline to the straight line of the aortic arch, gothic arch (**D**), romanesque arch (**E**), crenel arch (**F**).

Import CTA images into Philips IntelliSpace Portal V6.0 workstation post-processing software using thoracic macrovascular volume reproduction (VR), maximum density projection (MIP), and other image post-processing techniques, so that the ascending aorta, aortic arch, descending aorta and thoracic aorta transverse septum are at the maximum level in the oblique sagittal position. The maximum inner diameter of the ascending aorta (AOA) at the level of the main pulmonary artery, the maximum inner diameter of the proximal arch (D1), the distal arch (D2), and the isthmus (D3), the middle section of the descending aorta (D4) and the descending aorta at the transverse septum (D5) were measured. AAO-DAO angle, TAO-DAO angle, aortic arch height (A), and aortic arch width (T) were measured in the way shown in Figure 1, and the arch morphology was defined. Two senior pediatric imaging physicians engaged in cardiovascular disease research measured the images blinded, and agreed upon disagreement; all measurements were carried out 2 times and then take the average. According to the diagnostic criteria proposed in the Chinese expert consensus on the surgical treatment of congenital heart disease, D1/AOA, D2/AOA, and D3/AOA were calculated to evaluate the development of the aortic arch, and D4/AOA, D5/AOA and the height-to-width ratio (A/T) were calculated.

### 2.5. Computational fluid dynamics technology

**Figure 2:**
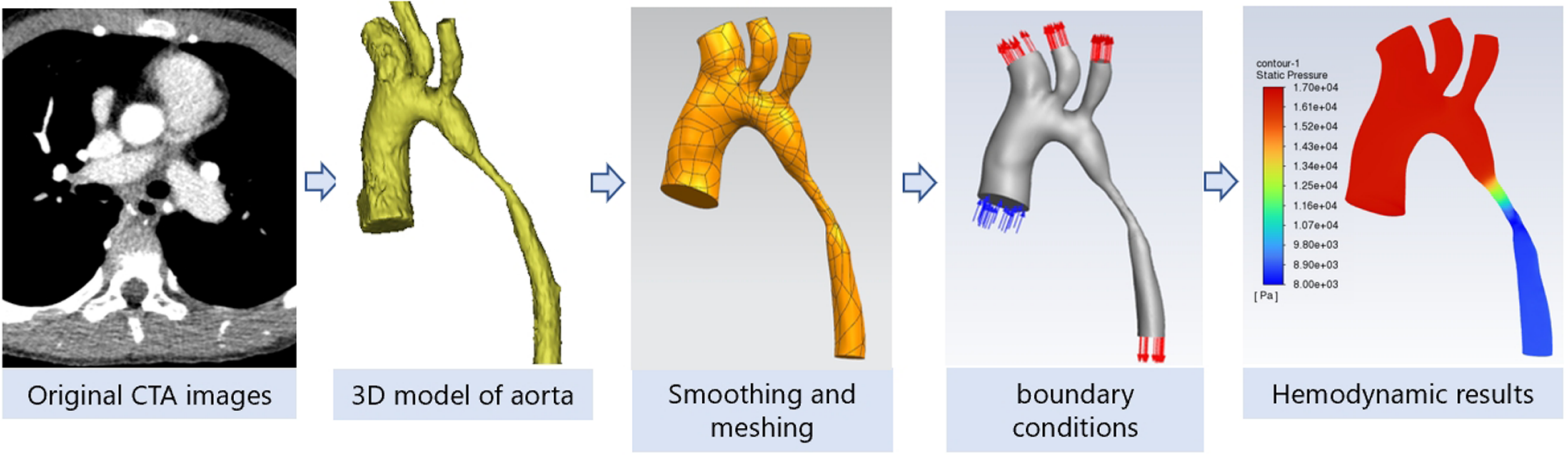
Computational fluid dynamics technical process.

(1) Image reconstruction: the original CTA images (DICOM format) of the patient were imported into Mimics software, by adjusting the threshold range to fit the blood in the heart and aorta, and editing the mask many times to preliminarily construct a three-dimensional model of the aorta. The aortic starts from 0.5 cm above the aortic valve to the descending aortic perforating diaphragm, and the three aortic branches, including the brachiocephalic trunk, the left common carotid artery (LCCA) and the left subclavian artery (LSCA), are retained, and then the model is smoothed and meshed in 3-matic software to save the model. (2) Setting boundary conditions and establishing hemodynamic model: the blood vessel wall is assumed as a rigid wall without slip, the blood is an insulated incompressible Newtonian fluid, the density is 1,050 kg/m^3^, the kinematic viscosity coefficient is 0.0035 Pa·s, and the flow mode is laminar flow. The inlet (0.5 cm above the aortic valve) border has a velocity 1.2 m/s, measured by TTE. The puncture pressure of the left upper limb is the outlet boundary of the brachiocephalic trunk, LCCA and LSCA, and the puncture pressure of the left lower limb is the exit boundary of the descending aorta. (3) Flow simulation: using Fluent software to solve the fluid flow, the conservation of mass (continuity equation) and the conservation of dynamics (Navier-Stokes) partial differential equation of fluid flow and control of blood flow by finite volume method, set the cardiac period to 0.8 s. The calculation time step is 0.005 s, and the maximum number of iterations in each time step is 200; (4) The convergence calculation results are extracted, the calculation results of the last cycle are extracted, and post-processed in CFD-Post software to output the systolic peak blood flow pressure along the central axis from the beginning of the aorta to the descending aortic diaphragm. The above process is shown in Figure 2.

### 2.6. Statistical analysis

SPSS26 software was used for statistical analysis. Quantitative data are expressed as mean ± standard deviation (*M* ± SD) and qualitative data are expressed as frequency (%). The normal distribution test uses the Kolmogorov-Smirnov method, and the homogeneity of variance test uses the Levene test. The *t*-test or *χ*^2^ test was used for comparison between groups, and the difference was statistically significant at *P* < 0.05.

## 3. Results

According to the arch morphology, 95 patients were divided into gothic arch (*n* = 27, 28.4%), crenel arch (*n* = 25, 26.3%), and romanesque arch (*n* = 43, 45.3%) (Table 1). There was no significant difference in clinical data such as gender, age, weight, and PSPG in the three groups (*P* > 0.05). The morphological measurements of CTA in three groups of patients are shown in Figure 1. There were no significant differences between D1/AOA and D2/AOA among gothic arch, crenel arch, and romanesque arch (*P* > 0.05). The differences in D3/AOA, D4/AOA, and D5/AOA among the three groups were statistically significant (*P* < 0.05), D4/AOA, D5/AOA of the gothic arch group were smaller than the crenel arch group (*P* < 0.05), and the D3/AOA and D5/AOA of gothic arch group were smaller than the romanesque arch group (*P* < 0.05). The difference in AAO-DAO angle among the three groups was statistically significant (*P* < 0.05), the AAO-DAO angle of gothic arch was smaller than that of romanesque arch and crenel arch group (*P* < 0.05). There was no significant difference in the TAO-DAO angle between the three groups (*P* > 0.05). The difference in A/T values among the three groups was statistically significant (*P* < 0.05). The average A/T values in the gothic arch, crenel arch, romanesque arch group were 0.70148 ± 0.014898, 0.49028 ± 0.013728, 0.58212 ± 0.012791, respectively. The A/T values: gothic arch > romanesque arch > crenel arch (*P* < 0.05).

**Table 1:** Comparison of clinical data and CT morphological data of the three groups (*M* ± SD).

Project	Gothic arch	Crenel arch	Romanesque arch	*χ*^2^/*t* value	*P* value
Gender (*M*, %)	12 (44.4%)	17 (68.0%)	25 (58.1%)		>0.05
Average age (month)	14.3444 ± 5.64625	5.0307 ± 1.93247	6.7548 ± 1.78188	2.096	>0.05
Average weight (kg)	6.996 ± 0.9809	4.808 ± 0.5305	5.451 ± 0.4003	2.754	>0.05
PSPG (mmHg)	38.585 ± 3.5754	43.076 ± 3.4719	38.557 ± 2.4324	0.742	>0.05
PDA (*n*, %)	19 (70.4%)	18 (72%)	35 (83.3%)	0.670	>0.05
D1/AOA	0.6304 ± 0.02607	0.6923 ± 0.03201	0.6646 ± 0.02213	1.168	>0.05
D2/AOA	0.5344 ± 0.02710	0.5614 ± 0.02282	0.5634 ± 0.02092	0.440	>0.05
D3/AOA	0.2896 ± 0.02266*	0.3078 ± 0.02511	0.3764 ± 0.01492*	3.692	<0.05
D4/AOA	0.7098 ± 0.02982**	0.8404 ± 0.04272**	0.7729 ± 0.02893	3.155	<0.05
D5/AOA	0.6414 ± .02788*^,^**	0.7731 ± 0.04082**	0.7292 ± 0.02083*	4.725	<0.05
AAO-DAO	26.74 ± 1.578*^,^**	34.56 ± 1.791**	31.88 ± 0.941*	7.321	<0.05
TAO-DAO	109.81 ± 1.716	112.40 ± 2.106	108.57 ± 0.936	1.724	>0.05
A/T	0.70148 ± 0.014898***	0.49028 ± 0.013728***	0.58212 ± 0.012791***	47.107	<0.05

* There were significant differences between gothic arch and romanesque arch (*P* < 0.05).

** There was a significant difference between the gothic arch and the crenel arch group (*P* < 0.05).

*** Statistically significant difference between the three groups (*P* < 0.05).

**Figure 3:**
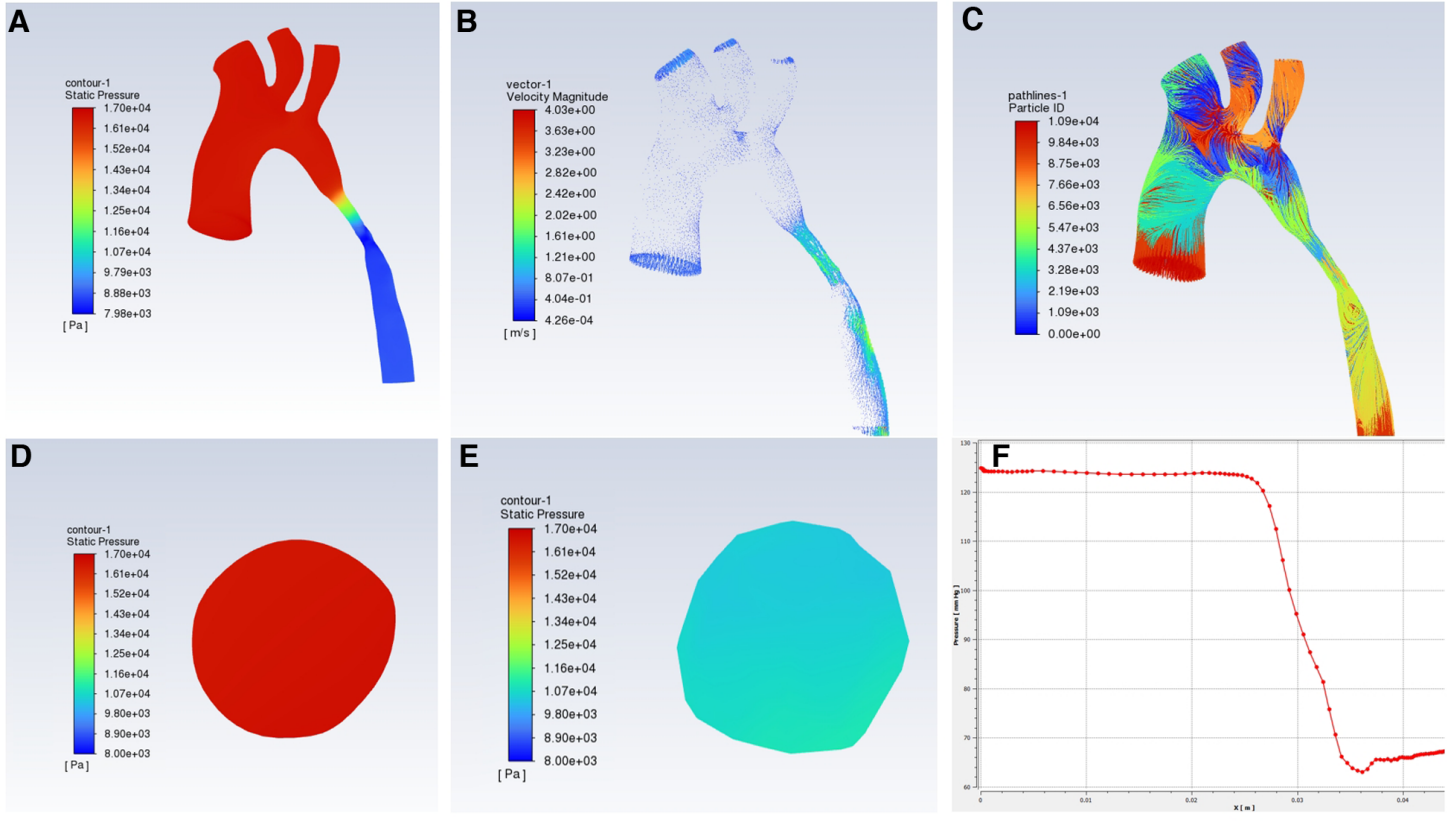
Hemodynamic changes in CFD in CoA patients with gothic aortic arch, (**A**) pressure distribution; (**B**) flow rate distribution; (**C**) Streamlined distribution; (**D**) pressure distribution at the ascending aorta level; (**E**) pressure distribution at the narrow level; (**F**) pressure curve.

The CTA image of one CoA child showed the gothic aortic arch and the hemodynamic changes of the aortic arch obtained by computational fluid dynamics calculations were shown in Figure 3. The pressure in the ascending aorta, arch, and brachiocephalic trunk, left common carotid artery, and left subclavian artery are high, and the pressure value does not decrease significantly. The pressure at the stenosis is drastically reduced, and the pressure difference between the distal stenosis and the descending aorta is about 58 mmHg, while the PSPG measured by TTE was 57.2 mmHg.

## 4. Discussion

CoA is a localized stenosis of the lumen of the aortic arch, which tends to occur at the level of the ductus arteriosus and the distal left subclavian artery. As a group of complex congenital heart diseases in children, it occurs slightly more in men than in women, accounting for about 6%–8% of all congenital heart diseases (1, 2). The pathogenesis of CoA is still unclear, there are several theories discussing the occurrence of CoA: (1) multiple genetic variants involved in cardiac angiogenesis and cardiac development, such as NOTCH1 genetic mutations could cause abnormal embryonic development; (2) The theory of catheter tissue migration holds that abnormal ductus arteriosus tissue in the aortic wall narrows the lumen of the aorta in the isthmus, and is further aggravated by the contraction and closure of the ductus arteriosus after birth; (3) Fetal hemodynamic theory believes that due to the presence of fetal ductus arteriosus, blood flow through the isthmus of the aorta is reduced, resulting in poor development and obvious fineness of the isthmus (2). Hypertension in the upper extremities and low blood perfusion in the lower extremities are characteristic of CoA, and stenosis leads to increased aortic pressure, aggravates left ventricular afterload, could induce myocardial hypertrophy, and eventually evolves into chronic heart failure (12). CoA has a poor natural prognosis, with approximately 5% of patients dying within the first few weeks, and approximately 10% of patients aged 1-6months occurs heart failure, and the median survival age is only 31 years old (13).

Many studies have pointed out that clinical consequences such as hypertension are related to abnormal aortic arch morphology (14). According to the shape of the aortic arch, it can be defined as gothic, crenel, or romanesque arch, which comes from the corresponding architectural style. They are triangular, rectangular, and round in shape respectively. Normally the aortic arch is generally romanesque (9). Among the CoA cases included in our study, romanesque arches accounted for the majority, some were crenel arches, and gothic arches showed a high proportion, suggesting that the occurrence of CoA may affect the arch morphological structure of the aorta. Possibly because the above theory (2) points out, abnormal ductus arteriosus tissue contracts and pulls the aortic arch in the isthmus, causing the arch to be sharp or blunt, then the shape changes. The study of Sophocleous et al. (15) showed that as the height increases and the width decreases, the aortic arch curvature was greater, which correlated with the presence of CoA (*P* = 0.02). Ou et al. defined (8) defines arch morphology in terms of the height to the width ratio (A/T), and when A/T is greater than 0.8, it is defined as a gothic arch, A/T less than 0.6 is a crenel arch, and a romanesque arch in between. In this study, the difference in the A/T ratio between the three groups was statistically significant, but the mean A/T value of the gothic arch group did not reach more than 0.8, varying from Ou's study. It may be that the aortic arch in some children is sharp and triangular in shape, which could be clearly defined as a gothic arch. However, the aortic arch is poorly developed, showing a small height or width of the aortic arch. Also, the limitation caused by the small number of cases can not be excluded.

Several fetal cardiac ultrasound or magnetic resonance studies have proposed two new methods for measuring AAO-DAO angle and TAO-DAO angle (16). Arya et al. divided the patients into CoA and non-CoA groups by postnatal examination, and the results showed that the two angles obtained by prenatal echocardiogram measurement present significant differences between the two groups (17). The critical values were obtained: AAO-DAO angle ≤20.31° and TAO-DAO angle ≥96.15°, they two are novel indicators of accurate postnatal outcomes-presence or absence of CoA, making prenatal diagnosis of CoA more accurate and helping to improve delivery planning. In children, echocardiography could observe stenosis of the lumen of the aorta, the direction of blood flow, and measure the blood flow velocity at the narrowest part of the aorta and the pressure gradient across the stenosis. Cardiac magnetic resonance could show the aorta and its surrounding blood vessels in multiple directions and sequences without contrast agent, which is a non-invasive examination method, but it has high requirements for heart rate and respiration and children are not easy to cooperate, limiting its application. CTA is performed through powerful post-processing techniques, including maximum density projection (MIP), multiplanar reconstruction (MPR), and thoracic macrovascular volume reproduction (VR). These 2D/3D images show multi-plane, multi-angle intuitive stereoscopic imaging of CoA and provide accurate measurement of the length and degree of CoA. The collateral circulation and other malformations, as well as the position relationship between blood vessels and between blood vessels and soft tissues could be seen clearly (18, 19). Echocardiography and CTA complement each other to provide a strong reference for clarifying the diagnosis, location, and extent of CoA, and are more commonly used in children. This study is the first to propose the measurement of AAO-DAO angle and TAO-DAO angle in CoA children in CTA images. No significant difference was seen in the TAO-DAO angle among the three groups, and the results were similar to Wang's study (20). AAO-DAO angle exists differently when the arch morphology is different, and compared with the crenel arch and the romanesque arch, the AAO-DAO of the gothic arch is small and sharp. The AAO-DAO angles of the crenel arch are blunt. Thus, regarding the definition of the aortic arch morphology of CoA, in 2004 Phalla defined the gothic arch as an A/T ratio of >0.8. The definition of the height-to-width ratio of the three arch forms, the population turnover over the past 10 years, the need to establish a large database for contemporary children, combined with the subjective evaluation of arch morphology, the height-to-width ratio (A/T) and AAO-DAO angle, to re-obtain the guideline value finally.

Chinese experts such as Zhang a et al. (21) believed that aortic coarctation could be divided into simple CoA and Hypoplastic Aortic Arch (HAA). The diagnostic criteria of the latter commonly is that the diameter of the aortic near arch, distal arch, and isthmus is less than 60%, 50%, and 40% of the diameter of the ascending aorta, respectively. It is generally believed that the patient's imaging examination shows that the diameter of the aortic arch stenosis is ≤50% of the normal value, so the diagnosis is confirmed. In this study, the measurement data of CTA images were used to analyze the development of the aortic arch, and the D3/AOA ratios of the three groups are less than 0.40, yet CoA could be confirmed (22). There were no significant differences between the three groups of D1/AOA and D2/AOA, indicating that different arch morphologies had little effect on the development of the proximal and distal arches of the aorta. Due to the presence of stenosis, blood flow through the isthmus of the aorta and descending aorta is reduced, which could cause poor development of the aorta. In gothic arch, the D4/AOA and D5/AOA were smaller than that of the crenel arch group, and the D3/AOA and D5/AOA were smaller than that of the romanesque arch group, indicating that the gothic arch morphology had a greater adverse effect on the development of the aortic isthmus and descending aortic segment (23). Therefore, the gothic arch should be actively reconstructed during surgical repair of CoA, and similarly, if the stenosis is not corrected intraoperatively and the postoperative gothic arch is produced, the risk of severe postoperative hypertension will also be significantly increased, so the gothic arch should be avoided during surgery. Furthermore, previous studies of CoA have mostly measured the diameter of the aorta at all levels to calculate the degree of stenosis and the development of the aortic arch. But CoA is not only a disease with a narrowing aorta, it also causes dynamic changes. Clinically, malformed geometry is strongly correlated with hemodynamic complications. Several studies have pointed out that the incidence of hypertension in the gothic aortic arch is significantly higher than that of the crenel arch and the romanesque arch (24). The special arch morphology complicates the hemodynamic changes of the aortic arch.

Computational fluid dynamics (CFD) could show dynamic changes in the aortic arch. This technology reconstructs blood flow pipelines based on CTA, magnetic resonance angiography (MRA), or other imaging methods. CFD simulates blood flow and calculates flow rate, pressure, and other parameters to non-invasively evaluate hemodynamic changes. The 2022 American Heart Association (ACC)/American College of Cardiology (AHA) report states that TTE showing the peak systolic pressure gradient (PSPG) >20 mmHg based on upper extremity hypertension or left ventricular hypertrophy confirms the diagnosis of CoA (25). As shown in Figure 3, the aortic arch in this case is a gothic arch, with high pressure of about 125 mmHg from the ascending aorta to the distal of the stenosis, excessive blood supply to the brain and upper limb, sharp decrease in pressure at the stenosis, low blood pressure of the descending aorta, and insufficient blood supply to the lower body, according to which the clinical results such as oliguria and weakened femoral pulse could be explained from kinetics (26). The flow rate of the section of the aorta from ascending aorta to stenosis is uniform, about 1.2–1.7 m/s, and the blood flow is in a good laminar flow state. The flow rate of the stenosis and descending aorta segment increased significantly, and there was a velocity distribution greater than 2 m/s and different degrees of turbulence. Abnormal blood turbulence could increase aortic pressure drop, heart work, and even cause platelet aggregation and thrombosis. CFD simulation could be used to visually detect abnormal changes in the aorta of patients with CoA, such as abnormal blood flow velocity distribution, and abnormal turbulence, which is useful for providing relevant references to study the extent of CoA, the causes of associated complications, and the prognosis of treatment. Some studies have also proposed wall shear stress (WSS), a hemodynamic indicator acting on endothelial remodeling and arterial dilation (27). WSS leads to vascular inflammation, endothelial proliferation, and apoptosis imbalance through signal transduction, and uneven thickness of the tube wall, then aortic dilation, aneurysms, or atherosclerosis could occur (28, 29). Related studies have proposed that in CoA, WSS strongly depends on the geometry of the aortic arch, and the gothic arch has a higher WSS in the ascending aorta and transverse aorta, showing an eccentric distribution (30). Meanwhile, the sharp corners of the gothic arch could lead to greater pressure changes, which adversely affect the cardiovascular system. Increased aortic arch WSS causes structural changes in the aortic wall, increased stiffness, and decreased compliance, and long-term changes lead to progressive damage and pathological remodeling of vascular structure (31, 32), and eventually may lead to ascending thoracic aneurysms (ATAA) (33).

In addition, PSPG was correlated with the degree of stenosis (18). In this study, the 95 cases were grouped according to arch morphology, and there was no significant statistical difference in PSPG between the groups as measured by echocardiography. It was statistically found that 3 groups of CoA with patent ductus arteriosus (PDA) accounted for more than 70%. There was no significant statistical difference in the proportion of PDA among the three groups. In the presence of ductus arteriosus, the blood flow discharged from the right ventricle could enter the descending aorta through the pulmonary artery and PDA, supply lower extremity blood flow perfusion, and compensate for the effect of aortic arch stenosis on descending aorta dynamics in CoA patients. Lu et al. (34) included 36 patients with both CoA and PDA, of whom only 14 (38.9%) had upper arm-leg blood pressure difference (>10 mmHg). A pressure gradient >10 mmHg with significant collateral circulation could also be diagnosed CoA when the trans-aortic pressure gradient is not above 20 mmHg (25). The collateral circulation of CoA has abundant sources, which could originate from the internal mammary artery, intercostal artery, thyroid cervical trunk, vertebral artery, etc. (27). Agarwa et al. (35) reported a 5-year-old male with no obvious symptoms, presenting with a heart murmur, and TTE measured aortic isthmus blood flow velocity within the normal range, but abdominal aortic blood flow continued to be low, and blood pressure in upper and lower extremities was 139/87, 72/48 mmHg, respectively. Further improve CTA examination to diagnose CoA with extensive vertebral collateral supplying the descending aorta, the diameter of the lumen of the surgically resected aortic isthmus is less than 1 mm. Thus, the velocity is measured by TTE, then calculating the pressure gradient generated by blood flow from the Bernoulli equation (36). The greater the velocity of blood flow to the aortic wall, the greater the pressure gradient between the ends of the stenosis. When stenosis is severe and persistent, the presence of collateral circulation blood flow may reduce blood flow through the aortic stenosis, and the pressure gradient measured by TTE may not be severe.

There are still certain limitations in this study: (1) the morphological definition of the aortic arch and the determination of the cut-off value need to be further explored and verified; (2) The exact clinical significance of CFD in CoA needs to be further analyzed and verified by more cases.

## 5. Conclusion

In summary, when the arch morphology of patients with aortic coarctation is gothic arch, the development of the aortic isthmus and descending aorta segment is often poor, which seriously affects the clinical consequences from morphology and kinetics. The evaluation of aortic arch morphology should be combined with subjective evaluation, height-to-width ratio, AAO-DAO angles, etc. At the same time, the application of computational fluid dynamics could visually display the hemodynamic changes in the aortic arch in CoA patients, which provides references for explaining comorbidities such as hypertension.

## Data Availability

The original contributions presented in the study are included in the article, further inquiries can be directed to the corresponding authors.
